# Emerging Technologies in Hand Orthopedic Surgery: Current Trends and Future Directions

**DOI:** 10.31661/gmj.v13i.3325

**Published:** 2024-04-16

**Authors:** Ahmad Dashtbozorg, Elaheh Heidarian, Malihe Sagheb Ray Shirazi, Zahra Movahednia, Maryam Jafari, Ramila Abedi Azar

**Affiliations:** ^1^ Department of Orthopedic Surgery, School of Medicine, Ahvaz Jundishapur University of Medical Sciences, Ahvaz, Iran; ^2^ Klinik für Unfallchirurgie und Orthopädie, Kinderorthopädie, Agaplesion Diakonieklinik Rotenburg, Rotenburg (Wümme), Germany; ^3^ Depertment of Anatomical Sciences, Faculty of Nursing and Midwifery, Hormozgan University of Medical Sciences, Bandar Abbas, Iran; ^4^ Department of Operating Room, Behbahan Faculty of Medical Sciences, Behbahan, Iran; ^5^ Department of General Surgery for Trauma, Shahid Beheshti University of Medical Sciences, Tehran, Iran; ^6^ Laboratory for Robotic Research, Iran University of Science and technology, Tehran, Iran

**Keywords:** Hand Orthopedic Surgery, Artificial Intelligence, Robotics, 3D Printing, Virtual Reality and Augmented Reality

## Abstract

Emerging technologies are changing hand surgery by improving surgical precision, minimizing tissue disruption, and expediting patient recovery. These advancements have the potential to revolutionize surgical procedures, patient outcomes, and rehabilitation processes. However, there are still challenges that need to be addressed before these technologies can be widely adopted. These challenges include the learning curve for surgeons, high costs, and ethical considerations. Future research should focus on addressing the limitations of these technologies, exploring their long-term effects, and evaluating their cost-effectiveness. To successfully implement them, a collaborative approach involving clinicians, researchers, engineers, and policymakers is necessary. This review provides an overview of current and future trends in emerging technologies for hand orthopedic surgery.

## Introduction

The emerging technologies significantly impact the field of orthopedic surgery [1]. The convergence of engineering innovation and medical expertise has given rise to a spectrum of emerging technologies that are reshaping the landscape of hand-surgical interventions. This surge in technological advancements holds the promise of revolutionizing not only surgical procedures but also patient outcomes, rehabilitation processes, and the overall trajectory of hand health [2][3].

Hand orthopedic surgery, which involves the diagnosis and treatment of disorders and injuries affecting the hand and upper extremities, has traditionally relied on manual techniques and limited tools [4]. Moreover, the role of emerging technologies in hand orthopedic surgery extends beyond the operating room. These advancements have also led to significant improvements in patient care and rehabilitation [5][6]. Virtual reality (VR) and augmented reality (AR) technologies, for instance, allow surgeons to simulate surgical procedures and educate patients about their condition [7][8][9]. This enhances patient understanding and enables more informed decision-making. Furthermore, emerging technologies have facilitated the development of personalized treatment plans and rehabilitation protocols, tailored to individual patients' needs and characteristics [10][11].

This technological advancements in hand orthopedic surgery have improved surgical outcomes and patient care and paved the way for further advancements. As technology continues to evolve, hand orthopedic surgery is expected to benefit from these advancements, ultimately leading to better patient outcomes and a higher quality of care [12][13].

In this review, we embark on a comprehesive exploration of the current trends and future directions in the realm of emerging technologies within hand and orthopedic surgery. From three-dimensional printing and robotics to augmented reality, wearable devices, and nanotechnology, these technologies are catalyzing a paradigm shift in the way hand surgeries are approached and executed. The convergence of precision, customization, and real-time data acquisition is unlocking new dimensions in the understanding and treatment of hand-related conditions.

## 1. Three-dimensional (3D) Printing in Hand Surgery

3D printing has revolutionized the field of hand surgery by allowing for the creation of custom implants and instruments tailored to each patient's unique anatomy [14][15][16].

This personalized approach has greatly improved surgical outcomes, particularly in cases of complex hand injuries and deformities [16][17].

The process begins with obtaining a detailed scan of the patient's hand using advanced imaging techniques such as CT scans or MRI [14]. These scans provide the necessary data to create a precise 3D model of the patient's hand, including any affected bones, tendons, or ligaments. Using this model, surgeons can design and print custom implants that perfectly fit the patient's anatomy, leading to improved functionality and reduced risk of complications [14][15][16][17][18].

3D printing also offers the flexibility to create surgical tools and guides that are specifically adapted to the intricacies of each case. This level of customization ensures that surgeons have access to the most suitable instruments and aids in achieving optimal results during procedures [19][20][21].

1.1. Patient-specific Implants

Traditional implants often face challenges in achieving an ideal fit due to anatomical variations among individuals. However, with 3D printing, implants can be precisely tailored to the unique anatomy of each patient [22][23].

This customization not only enhances the implant's fit but also optimizes its functionality, leading to improved postoperative outcomes and a reduction in complications. The use of 3D printing for patient-specific implants has shown promising results in hand surgery, and further research is needed to explore its long-term effects [15][18][24].

1.2. Anatomical Models for Preoperative Planning

The creation of detailed anatomical models through 3D printing has become an invaluable asset in preoperative planning for hand surgeries [15]. Surgeons can now visualize and interact with patient-specific anatomical structures, allowing for a more comprehensive understanding of complex cases [14]. This hands-on approach aids in the formulation of precise surgical strategies, ultimately contributing to enhanced procedural accuracy and improved patient outcomes. The use of 3D printed anatomical models has shown potential in reducing surgical complications and improving surgical efficiency [15][24].

1.3. Custom Prosthetics

The field of prosthetics has witnessed a transformative shift with the advent of 3D printing technology. Custom-designed prosthetic hands, fingers, or other orthotic devices can be manufactured with meticulous precision, accommodating the specific needs and preferences of individual patients [14][15]. This personalization not only improves the comfort and functionality of prosthetics but also addresses the aesthetic concerns of patients, fostering a more holistic approach to rehabilitation. The use of 3D printing for custom prosthetics has shown promising results in improving patient satisfaction and quality of life [25][26].

## 2. Robotic-assisted Systems

Robotic systems are designed to enhance surgeon ergonomics, reducing fatigue during lengthy procedures. Improved ergonomics contribute to sustained focus and steady control, ultimately enhancing surgery [27][28]. Robotic-assisted hand surgery offers several advantages over traditional open surgeries. One significant advantage is improved surgical precision. Robotics enables surgeons to execute precise movements with sub-millimeter accuracy, which is particularly beneficial in procedures requiring delicate manipulations such as nerve repair or microsurgical interventions. This level of precision can lead to better outcomes and reduced risk of complications [29][30].

Another advantage of robotic-assisted hand surgery is minimized tissue trauma. Minimally invasive robotic procedures often result in reduced tissue trauma compared to traditional open surgeries. This can translate to less postoperative pain, faster recovery times, and improved patient satisfaction. By minimizing tissue trauma, robotic-assisted hand surgery offers a less invasive option for patients, leading to improved overall patient experience [31][32]. Additionally, robotic systems are designed to enhance surgeon ergonomics. The ergonomic design of robotic systems reduces fatigue during lengthy procedures, allowing surgeons to maintain focus and steady control. This ultimately contributes to enhanced surgical outcomes. By reducing fatigue and improving ergonomics, robotic-assisted hand surgery can improve the overall quality of care provided to patients [33][34].

Despite the advantages of robotic-assisted hand surgery, there are certain considerations to use this technology. The integration of robotic systems in hand surgery has introduced technological complexities that require specialized training for surgeons and support staff [35][36][37].

Overcoming these challenges necessitates ongoing education and the development of standardized protocols. Surgeons must familiarize themselves with the robotic platform, master the hand-eye coordination required for precise manipulation, and adapt to the unique interface and feedback mechanisms provided by the robotic system [33][38].

Furthermore, support staff, including nurses and technicians, need to be trained to effectively assist in robotic-assisted procedures. These training requirements highlight the need for continuous education and collaboration between healthcare professionals and technology developers. This collaboration is essential for successful implementation and advancement of robotic-assisted hand surgery in the healthcare field [36][38][39].

Cost considerations also pose challenges to the widespread adoption of robotic-assisted hand surgery. The initial investment in robotic systems, including the purchase of the equipment and installation, can be substantial [31]. Additionally, maintenance costs, including regular servicing and software updates, need to be factored into the overall budget. Furthermore, the need for dedicated personnel to operate and maintain the robotic system adds to the financial burden. Despite these challenges, studies have shown that the benefits of robotic-assisted hand surgery, such as reduced surgical time, improved accuracy, and enhanced patient outcomes, can potentially offset the initial investment and ongoing costs [40][41].

Currently, robotic-assisted hand surgery finds applications in various procedures, including arthroplasty and peripheral nerve surgeries [42][43]. These procedures benefit from the precision and dexterity offered by robotic systems, leading to improved surgical outcomes and reduced post-operative complications [30][44].

## 3. Augmented Reality (AR) and Virtual Reality (VR)

AR and VR are rapidly evolving technologies that overlay or replace the physical world with digital information [9][10]. In recent years, these technologies have been increasingly integrated into various medical disciplines, including hand surgery. The use of AR and VR in hand surgery provides a distinctive opportunity to revolutionize conventional surgical planning, education, and intraoperative guidance, ultimately improving procedural accuracy and patient outcomes [7][9][10].

These technologies have been used to create preoperative surgical plans, enabling surgeons to visualize the surgical site from various perspectives [7][10]. The use of AR and VR in surgical planning can enhance a surgeon's understanding of a patient's unique anatomy and pathology, leading to a more precise and personalized surgical approach. Vles et al. [10] presented that these technology can reduce operative time and improve surgical precision, resulting in better patient outcomes.

Additionally, AR and VR provide an innovative platform for surgical education due to their immersive nature [9][37]. Trainees can practice surgical procedures in a virtual environment, improving their technical skills and decision-making abilities. These technologies provide real-time feedback, enhancing the learning process. Studies have shown that AR and VR-based training can improve trainees' procedural accuracy and confidence, thereby enhancing patient safety [45].

Moreover, AR and VR technologies have been used to provide real-time, intraoperative guidance, enhancing the surgeon's spatial awareness and precision [7]. Digital information, such as the patient's unique anatomy or the planned surgical approach, is overlaid onto the surgical field to assist the surgeon during the procedure [7][10].

## 4. Smart Implants and Wearable Technologies

The smart implants and wearable technologies offer real-time monitoring of surgical sites, providing clinicians with immediate insights into postoperative conditions such as temperature, inflammation, and healing progress [13][46][47][48]. Biomechanical sensors embedded in implants can monitor joint motion and muscle activity, providing valuable feedback on the effectiveness of surgical interventions and guiding personalized rehabilitation strategies [46][49].

Wearable devices extend post-operative monitoring beyond the clinical setting, including smartwatches and specialized rehabilitation wearables [50]. They provide a comprehensive understanding of the patient's recovery journey by continuously tracking vital signs, activity levels, and rehabilitation exercises. These devices also promote increased patient engagement through features such as real-time feedback, reminders, and personalized rehabilitation plans, resulting in improved adherence to postoperative care regimens and better outcome [51][52].

Large datasets related to postoperative recovery are being generated through the integration of smart implants and wearables [53]. Advanced analytics and machine-learning algorithms can process this data, providing clinicians with valuable insights into recovery trends, potential complications, and individualized responses. Real-time data collection also enables early detection of complications, allowing for timely interventions and improved patient safety [13][52]. Smart implants and wearables allow for personalized rehabilitation plans based on real-time data, optimizing recovery and promoting functional restoration [49][52].

By enabling remote monitoring through telehealth platforms, wearable technologies also extend the reach of post-operative care. Clinicians can assess patient progress, provide guidance, and make necessary adjustments to rehabilitation plans without the need for frequent in-person visits. This remote monitoring capability improves patient convenience and reduces the burden of travel, while still ensuring effective postoperative care [48][51][52].

## 5. Biomechanics and Sensor Technologies for Hand Function Assessment

The human hand has a complex biomechanical architecture that performs a multitude of tasks with precision and dexterity [11]. Assessing its function after surgical intervention is a critical component of patient care. Recent advancements in sensor technologies have opened new avenues for the detailed evaluation of hand function, providing valuable data that can inform treatment and rehabilitation strategies [54].

The use of sensor technologies has revolutionized the collection of data on hand functions in real-time. This capability provides immediate feedback on a patient's performance during rehabilitation exercises, facilitating more dynamic and responsive treatment plans [46][54].

Additionally, the immediacy of data collection promotes greater patient engagement, as individuals can actively participate in their recovery by tracking their progress and setting tangible goals. Improved adherence to rehabilitation protocols is associated with enhanced patient engagement, resulting in better functional outcomes [49][55].

Sensor technologies, such as accelerometers, gyroscopes, and electromyography (EMG), enable precise measurement of joint kinematics and muscle activity, allowing for biomechanical assessment of joint movement and muscle activity [48]. These tools provide quantitative data that offer an objective basis for evaluating patient recovery. Clinicians can tailor rehabilitation programs to address specific deficits identified through sensor-based evaluations by assessing range of motion, force generation, and coordination [11][48][54].

## 6. Nanotechnology Applications

Hand orthopedic surgery involves procedures to restore the function and integrity of the hand's anatomical structures. The application of nanotechnology in this field offers unprecedented opportunities to improve the efficacy of surgical interventions [56]. Nanotechnology involves the manipulation of matter at the nanoscale, where unique phenomena enable novel applications. At this scale, materials exhibit physical, chemical, and biological properties that can be used to overcome the limitations of conventional hand orthopedic surgical approaches [57][58][59][60].

6.1. Drug Delivery Systems

Nanotechnology has had a transformative impact on the field of drug delivery systems by enabling precise control over the release of therapeutic agents at the intended site, thereby enhancing the effectiveness of drugs and minimizing adverse effects on the body as a whole [58]. Various nanocarriers, including liposomes, dendrimers, and polymeric nanoparticles, can be specifically engineered to transport anti-inflammatory, analgesic, or antimicrobial agents directly to the surgical site in the hand. This targeted approach offers significant advantages in the management of postoperative pain and infection, both of which are critical factors that can greatly influence surgical outcomes and patient recovery [59][58].

6.2. Tissue Engineering

Nanofibrous scaffolds, which include biocompatible and biodegradable materials, provide an ideal environment for cell attachment, proliferation, and differentiation [57]. So, this technology has greatly advanced the field of tissue engineering, specifically in the regeneration and repair of bone, tendons, and ligaments in hand surgery [61].

Tissue regeneration can be further enhanced by adding growth factors and other bioactive molecules to these scaffolds. Nanoscale surface modifications of scaffolds can imitate the natural extracellular matrix, promoting cellular interactions that lead to improved tissue integration and healing [62].

6.3. Implant Coatings

Hand orthopedic surgeries often involve the use of implants for fracture fixation or joint replacement [63]. Nanotechnology has been instrumental in developing coatings for these implants to improve biocompatibility, reduce wear, and prevent infection. Nanocoating can be designed to release antimicrobial agents, reduce inflammatory responses, and promote osteointegration. In addition, the longevity and success of the implant can be enhanced by the application of nanoscale surface textures that influence protein adsorption and cell behavior [56][62].

## 7. Challenges and Ethical Considerations

It is important to note that while nanotechnology offers promising applications in hand and orthopedic surgery, several challenges need to be addressed to translate these innovations into clinical practice [4]. The biocompatibility and long-term effects of nanomaterials must be thoroughly evaluated to ensure patient safety [56]. Additionally, manufacturing processes, scalability, and regulatory approvals present significant challenges [64]. Furthermore, the cost-effectiveness of these nanotechnological approaches must be considered in the context of healthcare economics [40][42][56].

Emerging technologies in the field of hand orthopedics present several challenges and ethical considerations that must be carefully considered. While the integration of new surgical techniques and tools offers significant advancements in patient care, the adoption of these technologies is accompanied by a complex array of obstacles. Addressing these challenges is crucial to ensure the smooth integration of innovation into clinical practice [56][65].

One of the major challenges associated with implementing emerging technologies in hand surgery is the significant learning curve for surgeons [66]. Mastery of new technologies requires extensive training and may result in a temporary decrease in a surgeon's efficiency, potentially affecting patient outcomes during the transitional period. Furthermore, the high cost of new equipment and the need for ongoing maintenance and updates can impose significant financial burdens on healthcare institutions [41][67][68].

Patient privacy is of paramount importance, particularly with the increasing use of digital health records and telemedicine. Surgeons and healthcare providers must ensure that all patient data is protected against unauthorized access and breaches, adhering to stringent confidentiality protocols [65][69].

Informed consent is another ethical imperative that must be rigorously upheld. Patients should receive comprehensive education on the risks and benefits of novel surgical interventions, including an explanation of the technology, potential outcomes, and alternative treatment options. This will enable them to make well-informed decisions regarding their healthcare [67][69].

The responsible use of technology is a major ethical concern that includes both patient safety and the careful implementation of new surgical techniques. Surgeons must evaluate the evidence supporting the use of emerging technologies objectively and balance innovation with the established principles of patient care [65].

## 8. Future Directions and Research Opportunities

Recent years have seen a rise in the use of cutting-edge technologies in hand orthopedic surgery, including robotics, artificial intelligence (AI), 3D printing, and VR. These advancements have the potential to improve surgical precision, speed up patient recovery, and reduce healthcare costs [14][22][24][53][70].

Looking to the future, the combination of AI and robotics has the potential to improve surgical procedures by providing a more precise and personalized level of care. Additionally, 3D printing technology could be used to create customized implants, which may lead to better patient outcomes [8][11][13][30].

Further research is needed in several areas concerning the use of AI and robotics in hand orthopedic surgery. Key areas of investigation include the safety and effectiveness of these technologies, requiring rigorous, large-scale clinical trials. Additionally, the ethical and legal implications of these advancements warrant exploration [65][71]. Studies should also assess the cost-effectiveness of AI and robotics and their potential impact on healthcare delivery and patient satisfaction [30][65][71][72].

A collaborative approach involving clinicians, researchers, engineers, and policymakers is needed to successfully implement these technologies. Innovation should be encouraged to address the limitations and challenges associated with these technologies [73][74][75]. Moreover, collaboration across disciplines could facilitate the development of new technologies and their integration into clinical practice [76].

Moreover, future research should focus on specific case studies that demonstrate the practical application of these ethical principles in clinical settings and hang surgery.

## Conclusion

The emergence of advanced technologies such as robotics, 3D printing, AR and VR, wearables, sensor technologies, and nanotechnology is poised to revolutionize hand orthopedic surgery, offering precision, customization, and data acquisition capabilities that are reshaping clinical practice and patient outcomes. Integration of robotics has enhanced surgical precision and minimized tissue trauma, while 3D printing has facilitated the creation of custom implants and tools, leading to improved outcomes. AR and VR technologies show promise in surgical planning and education, and wearables have transformed postoperative monitoring and rehabilitation. Sensor technologies enable detailed evaluation of hand function post-surgery, while nanotechnology holds potential for drug delivery and tissue engineering. Despite these advancements, challenges including the learning curve for surgeons, high costs, and ethical considerations remain. Future research should address these limitations and explore long-term effects and cost-effectiveness. Upholding ethical standards, including patient privacy and informed consent, is crucial for ensuring patient safety and trust. Collaboration among clinicians, researchers, engineers, and policymakers is essential for the successful implementation of these technologies.

## Conflict of Interest

None.

## References

[R1] Satava RM (2008). Advanced technologies and the future of medicine and surgery. Yonsei Med J.

[R2] Owens JG, Rauzi MR, Kittelson A, Graber J, Bade MJ, Johnson J, et al (2020). How New Technology Is Improving Physical Therapy. Curr Rev Musculoskelet Med.

[R3] Dupont PE, Nelson BJ, Goldfarb M, Hannaford B, Menciassi A, O’Malley MK, et al (2021). A decade retrospective of medical robotics research from 2010 to
2020. Sci Robot.

[R4] Chu CY, Patterson RM (2018). Soft robotic devices for hand rehabilitation and assistance: a
narrative review. J NeuroEngineering Rehabil.

[R5] Hakim RM, Tunis BG, Ross MD (2017). Rehabilitation robotics for the upper extremity: review with new
directions for orthopaedic disorders. Disabil Rehabil Assist Technol.

[R6] Croke L (Avai). Health care technology continues to improve patient care and work e.

[R7] Lungu AJ, Swinkels W, Claesen L, Tu P, Egger J, Chen X (2021). A review on the applications of virtual reality, augmented
reality and mixed reality in surgical simulation: an extension to different
kinds of surgery. Expert Rev Med Devices.

[R8] Verhey JT, Haglin JM, Verhey EM, Hartigan DE (2020). Virtual, augmented, and mixed reality applications in orthopedic
surgery. Int J Med Robot.

[R9] McKnight RR, Pean CA, Buck JS, Hwang JS, Hsu JR, Pierrie SN (2020). Virtual Reality and Augmented Reality—Translating Surgical
Training into Surgical Technique. Curr Rev Musculoskelet Med.

[R10] Vles MD, Terng NCO, Zijlstra K, Mureau MAM, Corten EML (2020). Virtual and augmented reality for preoperative planning in
plastic surgical procedures: A systematic review. J Plast Reconstr Aesthet Surg.

[R11] Kabir R, Sunny M, Ahmed H, Rahman M (2022). Hand Rehabilitation Devices: A Comprehensive Systematic Review. Micromachines.

[R12] Braun BJ, Grimm B, Hanflik AM, Marmor MT, Richter PH, Sands AK, et al (2020). Finding NEEMO: towards organizing smart digital solutions in
orthopaedic trauma surgery. EFORT Open Rev.

[R13] Chatterjee SK (2023). AN EVALUATION OF SMART IMPLANTS IN ORTHOPEDIC SURGERY THAT
ENHANCE PATIENT OUTCOMES. Student's Journal of Health Research Africa.

[R14] Keller M, Guebeli A, Thieringer F, Honigmann P (2021). Overview of In-Hospital 3D Printing and Practical Applications in
Hand Surgery Duan X, editor. BioMed Res Int.

[R15] Wixted CM, Peterson JR, Kadakia RJ, Adams SB (2021). Three-dimensional Printing in Orthopaedic Surgery: Current
Applications and Future Developments. JAAOS Glob Res Rev.

[R16] Zhang D, Bauer AS, Blazar P, Earp BE (2021). Three-dimensional printing in hand surgery. J Hand Surg.

[R17] Jacobo OM, Giachero VE, Hartwig DK, Mantrana GA (2018). Three-dimensional printing modeling: application in maxillofacial
and hand fractures and resident training. Eur J Plast Surg.

[R18] Galvez M, Asahi T, Baar A, Carcuro G, Cuchacovich N, Fuentes JA, et al (2018). Use of Three-dimensional Printing in Orthopaedic Surgical
Planning. JAAOS Glob Res Rev.

[R19] Aimar A, Palermo A, Innocenti B (2019). The Role of 3D Printing in Medical Applications: A State of the
Art. J Healthc Eng.

[R20] Hoang D, Perrault D, Stevanovic M, Ghiassi A (2016). Surgical applications of three-dimensional printing: a review of
the current literature & how to get started. Ann Transl Med.

[R21] Portnova AA, Mukherjee G, Peters KM, Yamane A, Steele KM (2018). Design of a 3D-printed, open-source wrist-driven orthosis for
individuals with spinal cord injury Gard SA, editor. PLOS ONE.

[R22] Diment LE, Thompson MS, Bergmann JHM (2017). Clinical efficacy and effectiveness of 3D printing: a systematic
review. BMJ Open.

[R23] Wong KC (2016). 3D-printed patient-specific applications in orthopedics. Orthop Res Rev.

[R24] Matter-Parrat V, Liverneaux P (2019). 3D printing in hand surgery. Hand Surg Rehabil.

[R25] Lee KH, Kim DK, Cha YH, Kwon JY, Kim DH, Kim SJ (2019). Personalized assistive device manufactured by 3D modelling and
printing techniques. Disabil Rehabil Assist Technol.

[R26] Alturkistani R, A K, Devasahayam S, Thomas R, Colombini EL, Cifuentes CA, et al (2020). Affordable passive 3D-printed prosthesis for persons with partial
hand amputation. Prosthet Orthot Int.

[R27] Kakar S (2017). What’s New in Hand and Wrist Surgery. JBJS.

[R28] Diana M, Marescaux J (2015). Robotic surgery. Br J Surg.

[R29] Ghandourah HSH, Schols RM, Wolfs JAGN, Altaweel F, Van Mulken (2023). Robotic Microsurgery in Plastic and Reconstructive Surgery: A
Literature Review. Surg Innov.

[R30] Chen AF, Kazarian GS, Jessop GW, Makhdom A (2018). Robotic Technology in Orthopaedic Surgery. JBJS.

[R31] Roh HF, Nam SH, Kim JM (2018). Robot-assisted laparoscopic surgery versus conventional
laparoscopic surgery in randomized controlled trials: A systematic review
and meta-analysis Dangal G, editor. PLOS ONE.

[R32] Kim M, Zhang Y, Jin S (2023). Soft tissue surgical robot for minimally invasive surgery: a
review. Biomed Eng Lett.

[R33] Higgins RM, Gould JC (Avai). Clinical Applications of Robotics in Gene.

[R34] Rodrigues Armijo, Huang CK, Carlson T, Oleynikov D, Siu KC (2020). Ergonomics Analysis for Subjective and Objective Fatigue Between
Laparoscopic and Robotic Surgical Skills Practice Among Surgeons. Surg Innov.

[R35] Kockerling F (Available from:
http://journal.frontiersin.org/article/10.3389/fsurg.2014.00015/abstract
).

[R36] Zhao B, Lam J, Hollandsworth HM, Lee AM, Lopez NE, Abbadessa B, et al (2020). General surgery training in the era of robotic surgery: a
qualitative analysis of perceptions from resident and attending surgeons. Surg Endosc.

[R37] Chowriappa A, Raza SJ, Fazili A, Field E, Malito C, Samarasekera D, et al (2015). Augmented-reality-based skills training for robot-assisted
urethrovesical anastomosis: a multi-institutional randomised controlled
trial. BJU Int.

[R38] SAGES Robotic, Chen R, Rodrigues Armijo, Krause C, Siu KC, Oleynikov D (2020). A comprehensive review of robotic surgery curriculum and training
for residents, fellows, and postgraduate surgical education. Surg Endosc.

[R39] Ali M, Phillips D, Kamson A, Nivar I, Dahl R, Hallock R (2022). Learning Curve of Robotic-Assisted Total Knee Arthroplasty for
Non-Fellowship-Trained Orthopedic Surgeons. Arthroplasty Today.

[R40] Pierce J, Needham K, Adams C, Coppolecchia A, Lavernia C (2020). Robotic arm-assisted knee surgery: an economic analysis. Am J Manag Care.

[R41] Shah NL, Laungani RG, Kaufman ME (Available from: http://link.springer.com/10.1007/978-3-319-91045-1_5
).

[R42] Kolessar DJ, Hayes DS, Harding JL, Rudraraju RT, Graham JH (Avai). Robotic-Arm Assisted Technology’s Impact on Knee Arthroplasty and
Associated Healt.

[R43] Chen LWY, Goh M, Goh R, Chao YK, Huang JJ, Kuo WL, et al (2021). Robotic-Assisted Peripheral Nerve Surgery: A Systematic Review. J Reconstr Microsurg.

[R44] Zhang F, Li H, Ba Z, Bo C, Li K (2019). Robotic arm-assisted vs conventional unicompartmental knee
arthroplasty: A meta-analysis of the effects on clinical outcomes. Medicine (Baltimore).

[R45] Yoo JS, Patel DS, Hrynewycz NM, Brundage TS, Singh K (2019). The utility of virtual reality and augmented reality in spine
surgery. Ann Transl Med.

[R46] Iyengar KarthikeyanP, Gowers BTV, Jain VK, Ahluwalia RajuS, Botchu R, Vaishya R (2021). Smart sensor implant technology in total knee arthroplasty. J Clin Orthop Trauma.

[R47] Ledet EH, Liddle B, Kradinova K, Harper S (2018). Smart implants in orthopedic surgery, improving patient outcomes:
a review. Innov Entrep Health.

[R48] Al-Ayyad M, Owida HA, De Fazio, Al-Naami B, Visconti P (2023). Electromyography Monitoring Systems in Rehabilitation: A Review
of Clinical Applications, Wearable Devices and Signal Acquisition
Methodologies. Electronics.

[R49] Iyengar KarthikeyanP, Kariya AD, Botchu R, Jain VK, Vaishya R (2022). Significant capabilities of SMART sensor technology and their
applications for Industry 4.0 in trauma and orthopaedics. Sens Int.

[R50] undefined undefined, undefined undefined (2023). Wearables for personalized monitoring of masticatory muscle
activity — opportunities, challenges, and the future. Clin Oral Investig.

[R51] de Fátima, Rosa V, Nepomuceno AC, Tavares C, Alberto N, Andre P, et al (2020). Wearable devices for remote physical rehabilitation using a
Fabry-Perot optical fiber sensor: ankle joint kinematic. IEEE Access.

[R52] Bowman T, Gervasoni E, Arienti C, Lazzarini SG, Negrini S, Crea S, et al (2021). Wearable devices for biofeedback rehabilitation: a systematic
review and meta-analysis to design application rules and estimate the
effectiveness on balance and gait outcomes in neurological diseases. Sensors.

[R53] Longo UG, De Salvatore, Candela V, Zollo G, Calabrese G, Fioravanti S, et al (2021). Augmented Reality, Virtual Reality and Artificial Intelligence in
Orthopedic Surgery: A Systematic Review. Appl Sci.

[R54] Jakob I, Kollreider A, Germanotta M, Benetti F, Cruciani A, Padua L, et al (Avai). Robotic and Sensor Technology for Upper Limb Reh.

[R55] Gassert R, Dietz V (2018). Rehabilitation robots for the treatment of sensorimotor deficits:
a neurophysiological perspective. J NeuroEngineering Rehabil.

[R56] Chen L, Zhou C, Jiang C, Huang X, Liu Z, Zhang H, et al (2023). Translation of nanotechnology-based implants for orthopedic
applications: current barriers and future perspective. Front Bioeng Biotechnol.

[R57] Annabi N, Tamayol A, Shin SR, Ghaemmaghami AM, Peppas NA, Khademhosseini A (2014). Surgical materials: Current challenges and nano-enabled
solutions. Nano Today.

[R58] Sindhu RK, Kaur H, Kumar M, Sofat M, Yapar EA, Esenturk I, et al (2021). The ameliorating approach of nanorobotics in the novel drug
delivery systems: a mechanistic review. J Drug Target.

[R59] Güven E (2021). Nanotechnology-based drug delivery systems in orthopedics. Jt Dis Relat Surg.

[R60] Deng Y, Zhou C, Fu L, Huang X, Liu Z, Zhao J, et al (2023). A mini-review on the emerging role of nanotechnology in
revolutionizing orthopedic surgery: challenges and the road ahead. Front Bioeng Biotechnol.

[R61] Leary SP, Liu CY, Apuzzo MLJ (2006). Toward the Emergence of Nanoneurosurgery: Part III—Nanomedicine:
Targeted Nanotherapy, Nanosurgery, and Progress Toward the Realization of
Nanoneurosurgery. Neurosurgery.

[R62] Li J, Esteban-Fernández De, Gao W, Zhang L, Wang J (2017). Micro/nanorobots for biomedicine: Delivery, surgery, sensing, and
detoxification. Sci Robot.

[R63] Yadav HKS, Alsalloum GA,  Al Halabi NA (Avai). Nanobionics and nanoengineered.

[R64] Pantalone D, Faini GS, Cialdai F, Sereni E, Bacci S, Bani D, et al (2021). Robot-assisted surgery in space: pros and cons A review from the
surgeon’s point of view. Npj Microgravity.

[R65] Sen RK, Tripathy SK, Shetty N (2023). Ethics in Clinical Orthopedic Surgery. Indian J Orthop.

[R66] Mahure SA, Teo GM, Kissin YD, Stulberg BN, Kreuzer S, Long WJ (2022). Learning curve for active robotic total knee arthroplasty. Knee Surg Sports Traumatol Arthrosc.

[R67] Moeini S, Shahriari M, Shamali M (2020). Ethical challenges of obtaining informed consent from surgical
patients. Nurs Ethics.

[R68] Bhimani SJ, Bhimani R, Smith A, Eccles C, Smith L, Malkani A (2020). Robotic-assisted total knee arthroplasty demonstrates decreased
postoperative pain and opioid usage compared to conventional total knee
arthroplasty. Bone Jt Open.

[R69] Haskell A, Kim T (2018). Implementation of Patient-Reported Outcomes Measurement
Information System Data Collection in a Private Orthopedic Surgery Practice. Foot Ankle Int.

[R70] Karimi A, HaddadPajouh H (2020). Artificial Intelligence, Important Assistant of Scientists and
Physicians. Galen Med J.

[R71] Hernigou P, Lustig S, Caton J (2023). Artificial intelligence and robots like us (surgeons) for people
like you (patients): toward a new human–robot-surgery shared experience What
is the moral and legal status of robots and surgeons in the operating room. Int Orthop.

[R72] Thurzo A, Kurilová V, Varga I (2021). Artificial Intelligence in Orthodontic Smart Application for
Treatment Coaching and Its Impact on Clinical Performance of Patients
Monitored with AI-TeleHealth System. Healthcare.

[R73] Maza G, Sharma A (2020). Past, present, and future of robotic surgery. Otolaryngol Clin North Am.

[R74] D’Souza M, Gendreau J, Feng A, Kim LH, Ho AL, Veeravagu A (2019). Robotic-Assisted Spine Surgery: History, Efficacy, Cost, And
Future Trends. Robot Surg Res Rev.

[R75] Bargar WL (2007). Robots in orthopaedic surgery: past, present, and future. Clin Orthop Relat Res.

[R76] Balasubramanian S, Klein J, Burdet E (2010). Robot-assisted rehabilitation of hand function. Curr Opin Neurol.

